# Disconnect between emergency contacts and surrogate decision-makers in the absence of advance directives

**DOI:** 10.1177/0269216312474486

**Published:** 2013-02-01

**Authors:** Mi-Kyung Song, Sandra E Ward

**Affiliations:** Adult and Geriatric Health Division, School of Nursing, University of North Carolina at Chapel Hill, Chapel Hill, NC, USA; School of Nursing, University of Wisconsin-Madison, Madison, WI, USA

**Keywords:** Surrogate decision-maker, emergency contact, end-of-life

## Abstract

**Background:**

The role played by emergency contacts can be extensive particularly for chronically seriously ill patients. If the patient's condition suddenly deteriorates, the emergency contact may be asked to make decisions that should instead fall to a designated surrogate decision-maker.

**Aims:**

To describe a process used to help chronically seriously ill patients identify a surrogate during study enrollment and to describe whether these surrogates were the same as the documented emergency contacts.

**Design:**

A descriptive cross-sectional study using eligibility assessment and baseline data from an efficacy trial. The parent trial tests the effects of an end-of-life communication intervention on patient and surrogate decision-maker outcomes, and thus, it was important to identify the surrogate. The study recruiter used a short battery of investigator-developed questions to help patients identify a surrogate.

**Setting/participants:**

Patients were 94 self-identified African Americans or Caucasians recruited from 18 outpatient dialysis centers, receiving dialysis for ≥6 months, with Charlson Comorbidity Index of ≥6 or 5 and hospitalized in the last 6 months.

**Results:**

When first approached, only three patients had a designated and documented surrogate. The remaining 91 selected a surrogate during the surrogate identification process. Of the 94 surrogates who were named, only 60 (63.8%) were also listed in the medical record as the emergency contact.

**Conclusions:**

In roughly one-third of instances, the selected surrogate was not the same person listed in official medical records as the emergency contact, which may pose potential problems in medical decision-making in the absence of advance directives.

## Introduction

Obtaining emergency contact information for patients being admitted to a health-care facility is a routine procedure. Yet little attention in practice and in the literature has been given to this process. Such lack of attention is surprising given that the role played by emergency contacts, while largely unknown, can be extensive given that they are the first to be contacted in the event of a sudden complication or deterioration in the patient's condition. In such situations, the emergency contact may be asked to make medical decisions that would otherwise fall to a designated surrogate decision-maker.

Several problems may arise if an emergency contact is asked to act as surrogate decision-maker, particularly in the absence of an advance directive. Most obviously, the emergency contact (even if he or she is the patient's next of kin) may not be a legitimate or recognized surrogate decision-maker. One obvious implication of such situations is that the emergency contact may not be prepared to act in a manner consistent with the patient's wishes.

Although the critical importance of advance care planning has been emphasized, the number of seriously chronically ill patients who make their wishes known (including naming a surrogate decision-maker) to family members and care providers has not improved except when a targeted intervention is used.^1,2^ Selecting a surrogate decision-maker is often an early step in advanced care planning.^3,4^ It requires that a patient think beyond who might be available as a contact and instead focus on the serious decisions in which that person might be involved. It is not at all clear that the person most conveniently able to serve as emergency contact would be the best surrogate decision-maker.

Here, we describe a process used to help chronically seriously ill patients identify a surrogate decision-maker as part of the enrollment process for a study, report the preferred surrogate decision-makers chosen by patients, and describe whether these surrogate decision-makers were the same as those persons named as emergency contacts.

## Methods

### Design and sample

This report is a descriptive cross-sectional study using eligibility assessment and baseline data from an ongoing efficacy trial of an end-of-life communication intervention. The parent study includes dyads of dialysis patients and their chosen surrogate decision-makers recruited from 18 outpatient dialysis centers in North Carolina. Patients were eligible for the trial if they were self-identified African American or Caucasian, receiving dialysis for at least 6 months prior to enrollment, and had a Charlson Comorbidity Index (CCI)^5^ score of ≥6 or 5 and hospitalized in the last 6 months (criteria associated with an estimated 30% one-patient-year mortality).^5–7^ A recruiter approached 125 patients who met these criteria to provide brief information about the study and to assess surrogate availability. Of those approached, 94 (75%) consented and completed baseline measures.

### Data collection and procedures

Procedures were approved by the Institutional Review Board of the University of North Carolina at Chapel Hill and the Office of Clinical Trials of the participating dialysis organizations. Because the parent study tests the effects of an intervention on patient and surrogate outcomes, it was critical to determine the appropriateness of the individual named as the surrogate; that individual had to be willing and able to serve as a surrogate decision-maker for the patient. The recruiter used a short battery of investigator-developed questions to help patients identify a surrogate decision-maker if they had not already done so.

The battery began with, “Please think about who should be your surrogate decision-maker or healthcare agent who should make medical decisions on your behalf if you can't make them yourself. You do not need to tell me the name now because there are certain things that I would like you to consider in deciding who should be your surrogate decision-maker.” Then, the remaining seven questions (Table 1) were asked. Finally, the patient was asked to state who his or her surrogate decision-maker should be.

The recruiter also collected patients' emergency contact information from medical records where such information had been obtained during the patient's admission to the dialysis center and then updated annually. If the emergency contact and the chosen surrogate decision-maker were different, the recruiter mentioned this to the patient and confirmed the surrogate designation. Sociodemographic data from patients included age, gender, race/ethnicity, years of formal education completed, marital status, annual household income, and surrogates' relationship to the patient.

## Results

Patients' (N = 94) mean age was 62.1 years (standard deviation (SD) = 12.4). The majority were Black (n = 69, 73.4%) and female (n = 56, 59.6%) and had completed at least a high school education (n = 79, 84%). Thirty-nine patients (41.5%) were married or living with a partner. Forty-one patients (43.6%) had an annual household income of US$13,000–US$29,000, and 27 patients (28.7%) reported it to be below US$13,000. The mean years on dialysis was 4.6 (SD = 4.7, median = 3.3). The mean CCI score was 7.76 (SD = 1.94); one-patient-year mortality was about 30%.^8^ The most common comorbid conditions were diabetes (84, 89.4%), congestive heart failure (52, 55.3%), peripheral vascular disease (39, 41.9%), cerebrovascular disease (33, 35%), and chronic pulmonary disease (28, 29.8%).

Of the 94 patients, only three already had a surrogate documented in an advance directive. The remaining 91 came to that decision after working through the guiding questions with the recruiter. Thirty-two patients (34%) named a spouse, 29 (30.9%) named a child, 17 (18.1%) named a sibling, 5 (5.3%) named a parent, 3 (3.2%) named a friend, and 8 (8.5%) named some other person (e.g. in-law or ex-wife). Of the 94 surrogates, 60 (63.8%) were also listed as the emergency contact whereas 34 (36.2%) were not.

**Figure 1:**
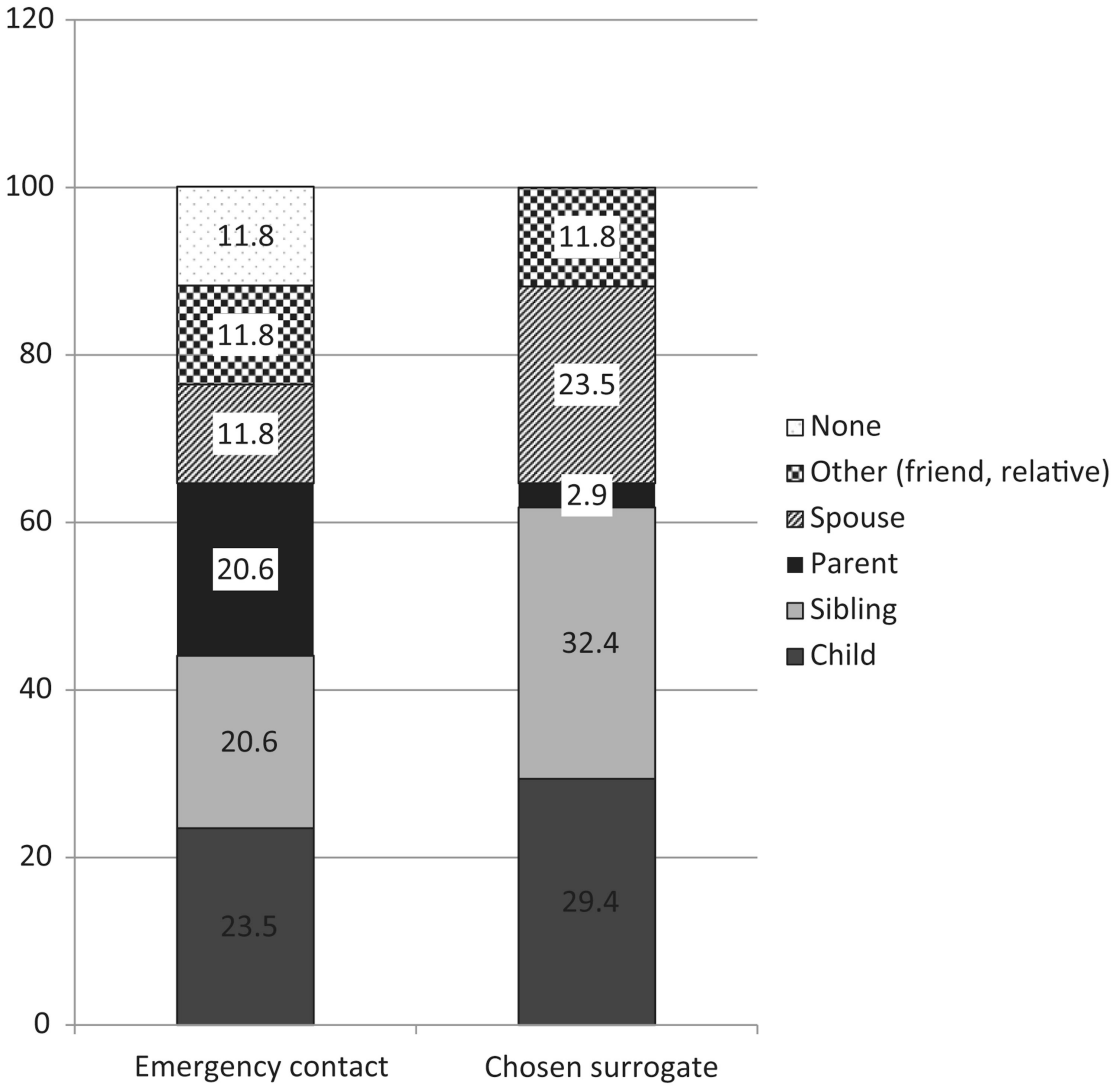
Emergency contact and surrogate designation for those 34 patients who had a discrepancy between them (numbers are in percentage).

Figure 1 presents the emergency contacts and surrogates of those 34 patients. There were 18 different combinations, such as a sibling was the emergency contact and a child was the surrogate. The most frequent disconnect was one child being an emergency contact and another child being a surrogate (n = 5, 14.7%) followed by one sibling being an emergency contact and another sibling being a surrogate (n = 4, 11.8%). There were no differences in sociodemographic characteristics between those who appointed a different person for their surrogate versus those who appointed the emergency contact as a surrogate.

## Discussion

Only three patients had identified a surrogate decision-maker before being recruited for the parent study. Once participants identified a surrogate, a sizable percentage (36.2%) of these surrogates was inconsistent with the emergency contact listed in the medical record. These findings reinforce the dismal state of affairs regarding the first step in advance care planning and establishing advance directives (having a willing, able, informed surrogate decision-maker) and call for a reconsideration of how little attention is given to the determination of emergency contacts. In the absence of a named surrogate decision-maker, many persons serving as emergency contacts may end up serving, de facto, as a decision-maker, even though neither the patient nor the emergency contact ever agreed to this designation.

The lack of assistance in determining a surrogate can be particularly problematic in states requiring notarization for an advance directive (e.g. North Carolina, South Carolina, and West Virginia), which requires that the document be signed in the presence of two witnesses and a notary public. This requirement is a barrier to formalizing surrogate designation for patients residing in those states and may foster a reliance on emergency contacts for decision-making.

Perhaps, it would be practical at the time of a patient's admission to help him or her identify a surrogate decision-maker rather than, or in addition to, identifying an emergency contact so that an appropriate individual would be available to be informed about the patient's medical conditions and treatment preferences and participate in decision-making if needed. Instead of the routine procedure of asking patients to name an emergency contact, the guiding questions used in the parent study could be easily used in any health-care setting to help patients identify a surrogate decision-maker. Such action would also set the stage for beginning advance care planning with both patient and surrogate.

## Figures and Tables

**Table 1:** Guiding questions used to identify surrogates.

Guiding questions
1. Is the person currently your primary caregiver or most likely to be the primary caregiver if you need one?
2. Is the person who you talk to often?
3. Is the person who knows and understands you well?
4. Is the person who you trust?
5. Is the person likely to be involved in medical decision-making for you?
6. Has the person been involved in medical decision-making for you in the past?
7. Can this person make decisions under very stressful situations?
